# Repressed Central Carbon Metabolism and Its Effect on Related Metabolic Pathways in Cefoperazone/Sulbactam-Resistant *Pseudomonas aeruginosa*

**DOI:** 10.3389/fmicb.2022.847634

**Published:** 2022-03-03

**Authors:** Yue-tao Chen, Ke-xin Yang, Zhen-yuan Dai, Huan Yi, Xuan-xian Peng, Hui Li, Zhuang-gui Chen

**Affiliations:** ^1^State Key Laboratory of Bio-Control, School of Life Sciences, Southern Marine Science and Engineering Guangdong Laboratory (Zhuhai), Guangdong Key Laboratory of Pharmaceutical Functional Genes, Sun Yat-sen University, Guangzhou, China; ^2^Department of Pediatrics, Department of Allergy, The Third Affiliated Hospital, Sun Yat-sen University, Guangzhou, China; ^3^Laboratory for Marine Fisheries Science and Food Production Processes, Qingdao National Laboratory for Marine Science and Technology, Qingdao, China

**Keywords:** *Pseudomonas aeruginosa*, antibiotic resistance, central carbon metabolism, cefoperazone/sulbactam, fatty acid metabolism, glutamate metabolism, riboflavin

## Abstract

Metabolic shift and antibiotic resistance have been reported in *Pseudomonas aeruginosa*. However, the global metabolic characteristics remain largely unknown. The present study characterizes the central carbon metabolism and its effect on other metabolic pathways in cefoperazone-sulbactam (SCF)-resistant *P. aeruginosa* (PA-R_SCF_). GC-MS-based metabolomics shows a repressed central carbon metabolism in PA-R_SCF_, which is confirmed by measuring expression of genes and activity of enzymes in the metabolism. Furthermore, expression of the genes that encode the enzymes for the first step of fatty acid biosynthesis, glutamate metabolism, and electron transport chain is reduced, confirmed by their enzymatic activity assay, and the key enzyme for riboflavin metabolism is also reduced, indicating the decreased metabolic flux to the four related metabolic pathways. Moreover, the role of the reduced riboflavin metabolism, being related to ROS generation, in SCF resistance is explored. Exogenous H_2_O_2_ potentiates SCF-mediated killing in a dose-dependent manner, suggesting that the decreased ROS resulted from the reduced riboflavin metabolism that contributed to the resistance. These results indicate that the repressed central carbon metabolism and related riboflavin metabolism contribute to SCF resistance, but increasing ROS can restore SCF sensitivity. These findings characterize the repressed central carbon metabolism and its effect on other metabolic pathways as the global metabolic features in PA-R_SCF_.

## Introduction

*Pseudomonas aeruginosa* is one of the most important opportunistic pathogens causing devastating acute nosocomial and chronic infections in individuals with compromised immune systems. Due to an outstanding capacity for being selected and for spreading antibiotic resistance, the infections caused by antibiotic-resistant *P. aeruginosa* pose a considerable threat regarding morbidity and mortality worldwide (Recio et al., 2020). One of the solutions to this crisis is to optimize the use of antibiotics that are currently available since antibiotic development pipeline runs dry (Talbot, 2010). In order to achieve this goal, it is necessary to have a comprehensive understanding of the resistant mechanisms.

*Pseudomonas aeruginosa* has intrinsic and acquired resistance to most antibiotics because of multiple chromosomal determinants as well as the complex regulatory pathways. These mechanisms that have the greatest effect on the higher natural resistance of *P. aeruginosa* compared to other Gram-negative bacteria include inducible AmpC cephalosporinase expression, constitutive (MexAB-OprM) and inducible (MexXY) efflux pump production, and low outer membrane permeability (Horcajada et al., 2019). However, further exploration is required for a deeper understanding of the antibiotic resistance mechanisms of *P. aeruginosa*.

Recent reports have indicated that metabolic environments are related to sensitivity to antibiotics (Peng et al., 2015; Ye et al., 2018a,b; Kuang et al., 2021a; Zhao et al., 2021). Bacterial metabolic states are one of the key determinants of antibiotic resistance (Jiang et al., 2020a; Zhang S. et al., 2020; Su et al., 2021). By profiling the metabolomes of *P. aeruginosa*, lipopolysaccharides, peptidoglycan, and phospholipid are decreased, which confer polymyxin resistance (Han et al., 2018, 2019). The synergistic killing by combining polymyxin and amikacin is linked to the disruption of cell envelope biogenesis and central carbohydrate metabolism, decreased levels of amino sugars, and a downregulated nucleotide pool (Hussein et al., 2019). Our recent report shows an endogenous NO-mediated cefoperazone-sulbactam (SCF) resistance and develops its reversion by metabolites in *P. aeruginosa* (Kuang et al., 2021b). However, the metabolic mechanisms of antibiotic resistance remain largely unknown.

SCF has better activities against *P. aeruginosa* and thereby is widely used in clinic (Sader et al., 2020; Ku and Yu, 2021). However, SCF-resistant *P. aeruginosa* strains are frequently isolated with the wide use of SCF (Huai et al., 2019), becoming a challenge to the conventional therapy. Since the central carbon metabolism is the core component of cell metabolism (Zhang Z. et al., 2020), it is key to understand the central carbon metabolism and its related metabolic pathways in SCF-resistant *P. aeruginosa* for further development of restoring SCF-mediated killing efficacy.

## Materials and Methods

### Bacterial Strains and Culture Conditions

*Pseudomonas aeruginosa* ATCC 27853 and clinically multidrug-resistant *P. aeruginosa* strains were from the collection of our laboratory, which were kept at −80°C for 6 months. Cefoperazone/sulbactam (SCF)-resistant *P. aeruginosa* (PA-R_*SCF*_) was selected from ATCC 27853 sequentially in LB medium plus 4 μg/ml SCF (1/2 minimum inhibitory concentrations, MIC). At the same time, LB medium without the antibiotic was used as a control as SCF-sensitive *P. aeruginosa* (PA-S). All bacteria strains were carefully stored at −80 °C. For experiments, strains were streaked from glycerol stocks on LB agar plates. Single clones were cultured overnight at 37°C with rigorous shaking in the tube containing 5 ml of LB medium. These overnight cultures were diluted to an OD_600_ of 1.6 and further diluted at a ratio of 1:100 or 1:1,000 into 50 ml of LB medium and incubated in 250-ml flasks at 37°C with shaking at 200rpm.

### Antibiotic Bactericidal Assay

Antibiotic bactericidal assay was performed as previously described (Kuang et al., 2021b). A single colony was propagated in LB broth at 37°C until OD_600_ reaches 0.25–0.3. The cultures were diluted to 1:1,000 using fresh LB broth and incubated for 16 h and harvested by centrifugation at 8,000 × *g* at 25°C for 3 min. The precipitates were washed three times and suspended in M9 medium until OD_600_ nm was 0.2 when H_2_O_2_ or/and SCF were added if desired, and then incubated at 37°C with shaking.

### Metabolic Profiling

Sample preparation was performed as previously described (Su et al., 2018). In brief, the overnight cultures of PA-S and PA-R_*SCF*_ were diluted to 1:100 in 50 ml of LB broth until OD_600_ nm was 1.0. The cultures were collected by centrifugation at 8,000 × *g* at 4°C for 3 min and immediately quenched with liquid nitrogen. The bacterial pellets were resuspended in pre-cooled PBS and adjusted to 1.0 of OD_600_ nm, and 10 ml of suspension cells was collected. Subsequently, 1 ml of pre-cooled methanol (HPLC grade) was immediately added to the pellet, and 10 μl of 0.1 mg/ml ribitol (Sigma) was added as internal quantitative standard. Metabolites were extracted by sonication for 10 min (35% power, 2 s pulse, 3 s pause) over ice-water mixture. After centrifugation, 800 μl of supernatant was removed and evaporated by a vacuum centrifuge dryer (Labconco, United States) at 37°C. Thereafter, 80 μl of 20 mg/ml methoxyamine hydrochloride (Sigma-Aldrich) in pyridine (Sigma-Aldrich) was added to resuspend the dried samples and incubated at 37°C for 3 h. Finally, 80 μl of *N*-methyl-*N*-(trimethylsilyl) trifluoroacetamide (MSTFA, Sigma) was added for derivatization for 30 min at 37°C. The derivatized sample with 1 μl was injected to DBS-MS column (230°C, 5 min). The column temperature was held at 85°C for 5 min, increased to 270°C at an increment of 15°C min^–1^, and then held at 270°C for 5 min. The carrier gas was helium, with a constant flow of 1ml/min. Mass spectra were acquired in the m/z range of 50–600. Metabolites were measured with a 7890A GC system (Agilent Technologies) combined with a 5975C VL MSD detector (Agilent Technologies). Four biological replicates with two technical replicas were performed for each strain.

### GC-MS Data Analysis

The statistical analysis was performed as previously described (Kuang et al., 2021a). Briefly, compounds were identified by matching them to data from the National Institute of Standards and Technology (NIST) library in the Xcalibur software (version 2.1). Each metabolite was expressed as peak area normalized to ribitol (internal standard) and the total intensity. Software IBM SPSS Statistics 17.0 was used for statistical analyses and differences were considered as significant if *p* < 0.05. Cluster analysis was performed with R software (R version 4.0.3). Principal component analysis (PCA) and orthogonal partial least squares-discriminant analysis (OPLS-DA) were conducted using SIMCA-P + (Version 12.0) software. Metabolic pathway enrichment analysis was conducted with MetaboAnalyst 5.0 computational platform^1^. Interactive Pathways (iPath) analysis was carried out by iPath3.0^2^. Data were plotted using GraphPad Prism version 8.0.

### Enzyme Activity Determination

Enzyme activity assays were performed as previously described (Kuang et al., 2021b). In brief, the overnight cultures were diluted to 1:100 using 50 ml of fresh LB broth and incubated until OD_600_ nm was 1.0, and harvested by centrifugation at 8,000 × *g* at 4°C for 3 min. Subsequently, the bacterial cells were washed three times and resuspended in 1 × PBS (pH 7.0), adjusted to OD_600_ nm at 1.0. Aliquots of 30 ml of cells were collected and resuspended in 600 μl of pre-cold PBS. The proteins were extracted with sonication for 6 min (35% power, 2 s pulse, 3 s pause) in ice-water mixture. After centrifugation, the supernatants were collected and quantified by a BCA protein concentration determination kit (Beyotime, P0009). Then, 300 μg of proteins was used for determination of malate dehydrogenase (MDH) and α-ketoglutarate dehydrogenase (KGDH) activity, and 500 μg of proteins was used for pyruvate dehydrogenase (PDH) and succinate dehydrogenase (SDH) activity. PBS was used as a blank control. The reaction buffer for PDH and KGDH includes 0.5 mM MTT, 2.5 mM MgCl_2_, 65 mM PMS, 0.2 mM TPP, and 80 mM sodium pyruvate/alpha-ketoglutaric acid potassium salt to a final volume of 200 μl in a 96-well plate. For SDH and MDH measurement, the reaction mixture is the same as above except that PMS was at 130 mM but without TPP. Subsequently, the plates were incubated in the dark at 37°C for 5 min for PDH/KGDH activity, at 37°C for 2 min for MDH activity, and at 37°C for 30 min for SDH activity. The reaction was quenched and read at 562 nm for colorimetric readings. In these cases, one unit of enzyme activity was defined as the quantity of enzyme that catalyzed the formation of 1 μmol of the corresponding product per minute at 37°C. Hexokinase (HK) and pyruvate kinase (PK) activity were measured with the HK or PK activity assay kit (Boxbio Science & Technology, China) respectively. 6-phosphogluconate dehydrogenase (6PGDH), glutamic-pyruvic transaminase (GPT), acetyl-CoA carboxylase (ACC), and complex I activity was measured, respectively, with a 6PGDH, GPT, ACC, and complex I activity assay kits obtained from Solarbio (Beijing Solarbio Science and Technology Co., Ltd., Beijing, China) according to the manufacturer’s instructions. Riboflavin kinase (RFK) activity was measured by enzyme-linked immunosorbent assay (ELISA) kit (Cloud-Clone Corp., United States) following the manufacturer’s protocol. Glucose 6-phosphate dehydrogenase (G6PDH) was measured by G6PDH assay kits (Suzhou Keming Biotechnology Co., Ltd., Suzhou, China), following the manufacturer’s protocol. Cytochrome c oxidase activity was measured using the bacterium EC1.9.3.1 colorimetry assay kit (GenMed Scientifics Inc., Shanghai, China, Cat No: GMS15029.1). All tests were repeated for at least three independent experiments.

### Quantitative Real-Time PCR

RNA extraction and quantitative real-time PCR (qRT-PCR) assays were performed as previously described (Kuang et al., 2021b). The bacterial samples used for RNA extraction were the same as those used for enzyme activity determination. Briefly, bacterial cells (1 ml; OD_600_ = 1.0) were harvested by centrifugation (12,000 × *g*, 4°C, 3 min) and immediately quenched by liquid nitrogen. Subsequently, total RNA was extracted by standard procedure using TRIzol Reagent (Invitrogen Life Technologies), chloroform, and isopropanol precipitation. RNA degradation and contamination were monitored on 1% agarose gels. Then, RNA concentration and purity were measured with NanoDrop One spectrophotometer (Thermo Fisher Scientific). For qRT-PCR analysis, total RNA samples (1 μg) were reverse-transcribed into cDNA using a PrimeScript RT reagent kit with gDNA Eraser (Takara, Japan) following the manufacturer’s protocol. The qRT-PCR was performed on a LightCycler 480 real-time PCR system (Roche) in 10-μl reaction mixtures using SYBR Green Premix (Takara, Japan). The cycling parameters were as follows: 95°C for 30 s followed by 40 cycles of 95°C for 5 s and annealing 60°C for 30 s (the primers are listed in Supplementary Table 1). Experimental RNA levels were normalized to 16S rRNA levels and each sample was analyzed in triplicate. The gene expression was calculated according to the 2^–ΔΔCt^ method.

### Measurement of ROS

ROS measurement was performed as previously described (Ye et al., 2018b). Bacteria were cultured as described above. Cells were resuspended in 1 ml of 0.85% NaCl and washed three times. Total intracellular ROS was determined by staining the cells with 2′,7′-dichlorfluorescin diacetate (Sigma, United States); 10^8^ cells were incubated with 10 μM 2′,7′-dichlorfluorescin diacetate (Sigma) for 30 min at 37°C in the dark. The samples were measured by using a microplate reader (Varioskan LUX, Thermo Scientific, United States) at an excitation wavelength of 485nm and an emission wavelength of 535nm. The *in vivo* ROS production was presented by calculating the fluorescence intensities of experimental groups (bacterial cells) deducted from the fluorescence in control groups (0.85% NaCl).

## Results

### Metabolome Is Altered in PA-R_SCF_

**FIGURE 1:**
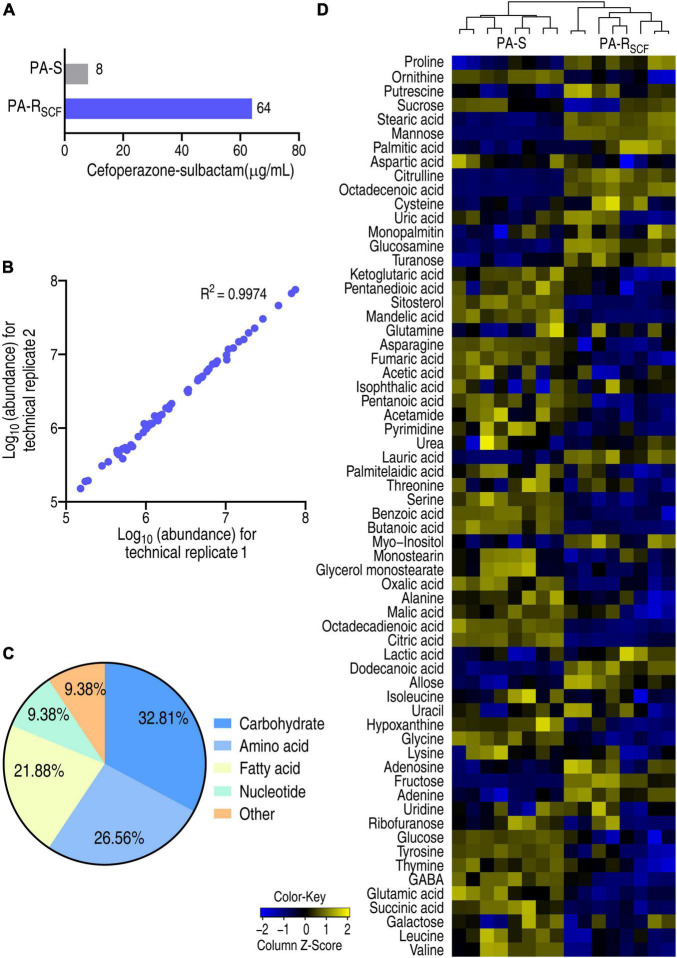
Resistance to SCF and metabolic profile in PA-R_SCF_. **(A)** MIC of PA-S and PA-R_SCF_ strains. **(B)** Abundance correlation of metabolites quantified in two technical replicates with high reproducibility. Metabolite abundance was represented in log_10_. **(C)** Categories of the metabolites in PA-S and PA-R_SCF_. **(D)** Heatmaps of unsupervised hierarchical clustering of differential metabolites in PA-S and PA-R_SCF_ (row). Blue and yellow indicate lower and higher expression of the metabolites relative to the mean and standard deviation of the row metabolite level, respectively (see color scale).

*Pseudomonas aeruginosa* ATCC 27853 strain was cultured in medium with or without 1/2 MIC (4 μg/ml) of SCF that generated PA-R_SCF_ and PA-S, respectively (Figure 1A). A GC-MS-based metabolomics approach was used to investigate a metabolic profile of the two strains. For each strain, four biological replicates with two technical replicas for each biological replicates were included, yielding a total of 16 data points. Correlation coefficients of two technical replicas ranked between 0.996 and 0.998, suggesting the high reproducibility of the data (Figure 1B). After removal of the internal standard (ribitol) and any known artificial peaks and integration of the same compounds, 64 metabolites with reliable signals were characterized for each sample. These metabolites were classified into 5 categories, carbohydrate (32.81%), amino acid (26.56%), fatty acid (21.88%), nucleotide (9.38%), and other (9.38%) (Figure 1C). PA-R_SCF_ and PA-S were separately clustered and displayed as a heatmap (Figure 1D). These results indicate that SCF induces a metabolic shift in PA-R_SCF_.

### Differential Metabolome Is Characterized in PA-R_SCF_

**FIGURE 2:**
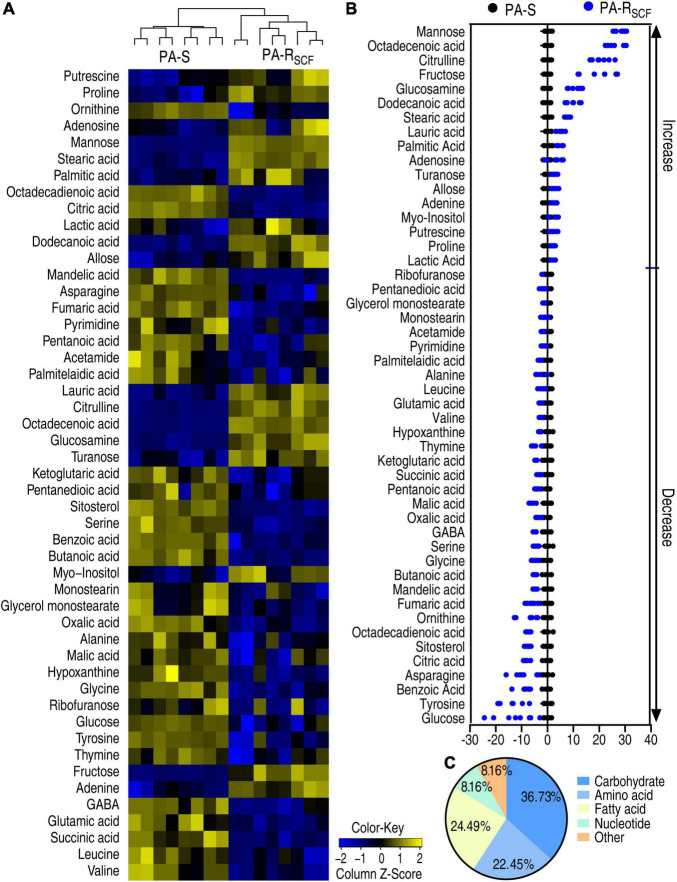
Differentially metabolic profiles in PA-R_SCF_. **(A)** Heatmaps of differential abundance of metabolites in PA-S and PA-R_SCF_ (row). Blue and yellow indicate lower and higher expression of the metabolites relative to the mean and standard deviation of the row metabolite level, respectively (see color scale). **(B)** *Z*-score plots of differential abundances of metabolites based on control. The data were separately scaled to the mean and standard deviation of the control (PA-S). Each point represents one metabolite in one technical repeat and colored by sample types. **(C)** Categories of differential abundance of metabolites in PA-S and PA-R_SCF_.

A Kruskal–Wallis test can determine whether or not there is a statistically significant difference between the medians of three or more independent groups. Thus, the test was used to identify differential abundances of metabolites in PA-R_SCF_ compared with PA-S. Among the 64 metabolites, 49 metabolites were different in abundance in PA-R_SCF_ (*p* < 0.05) and displayed as a heatmap (Figure 2A). *Z*-score, describing the position of a raw score in terms of its distance from the mean, exhibited metabolite variation between −12.43 and 28.20, with 17 increased and 32 decreased metabolites in PA-R_SCF_ (Figure 2B). The differential abundances of metabolites were classified into 5 categories, carbohydrate (36.73%), amino acid (22.45%), fatty acid (24.49%), nucleotide (8.16%), and other (8.16%) (Figure 2C), demonstrating that the carbohydrates, amino acids, and fatty acids were largely influenced in PA-R_SCF_.

### Metabolic Pathways Are Enriched in PA-R_SCF_

**FIGURE 3:**
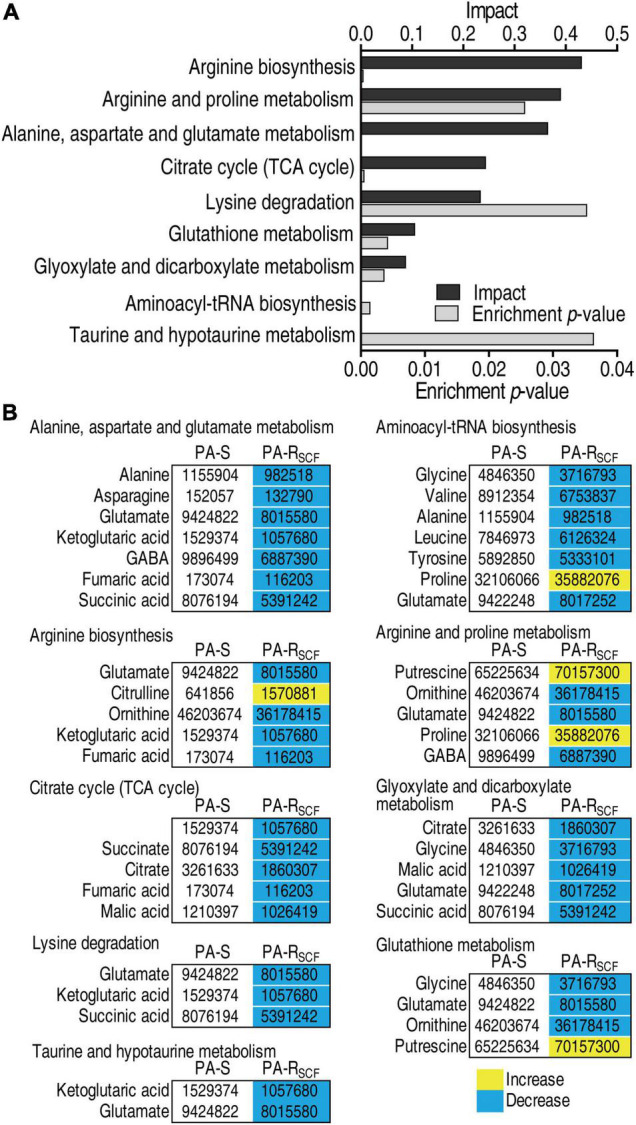
Enrichment of metabolic pathways in PA-R_SCF_. **(A)** Pathway enrichment of differential abundance of metabolites in PA-S and PA-R_SCF_. Significantly enriched pathways are selected to plot. *p* < 0.05. **(B)** Integrated analysis of pathways enriched by significantly associated metabolites. Yellow color and blue color indicate increased and decreased metabolites, respectively.

All substrates are chemically transformed in reactions that belong to pathways, essential for the correct functioning of a biological system. Thus, metabolites of differential abundance between PA-S and PA-R_SCF_ were analyzed for pathway enrichment. A total of 9 metabolic pathways were enriched in PA-R_SCF_. Arginine biosynthesis; arginine and proline metabolism; alanine, aspartate, and glutamate metabolism; the TCA cycle; and lysine degradation were listed as the first five impacted pathways (Figure 3A). Importantly, all metabolites detected in alanine, aspartate, and glutamate metabolism; the TCA cycle; glyoxylate and dicarboxylate metabolism; lysine degradation; and taurine and hypotaurine metabolism were decreased (Figure 3B) as compared to PA-S. Among them, the TCA cycle is located in the central carbon metabolism and alanine, aspartate, and glutamate metabolism fuels the TCA cycle, while the TCA provides a source for taurine and hypotaurine metabolism. These data suggest that the TCA cycle is impaired. A recent report has indicated that the pyruvate cycle (the P cycle) instead of the TCA cycle provides respiratory energy (Su et al., 2018). Therefore, the reduced P cycle is a significant metabolic feature of PA-R_SCF._

### Crucial Biomarkers Are Identified and Global Metabolism Is Viewed in PA-R_SCF_

**FIGURE 4:**
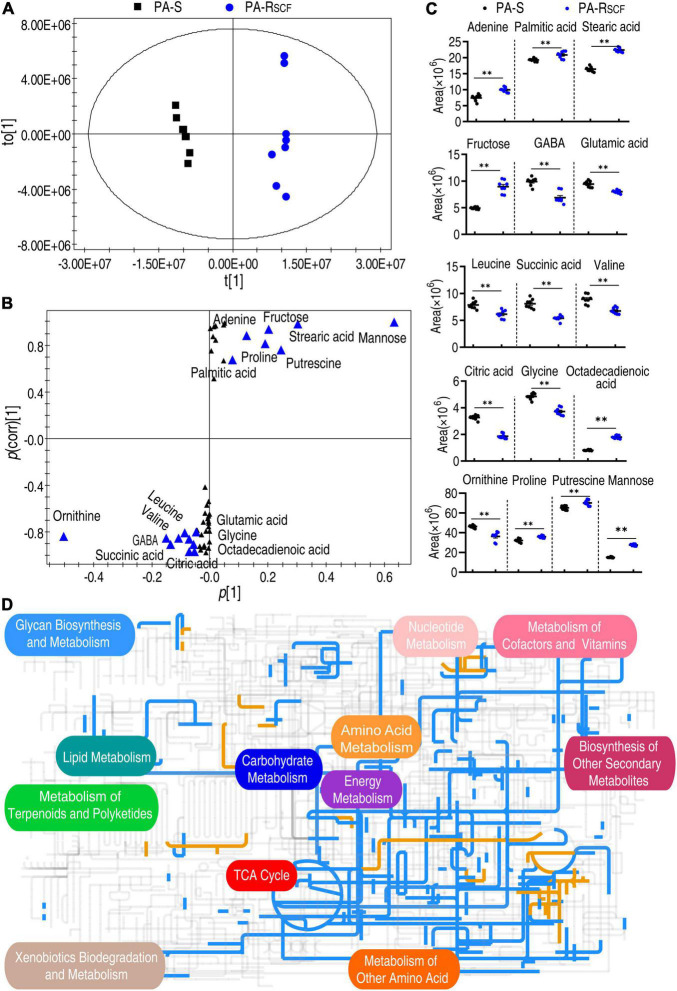
Identification of biomarkers and iPath analysis in PA-R_SCF_. **(A)** The PCA analysis between PA-S and PA-R_SCF_. Each dot represents the technique replicas in the plot. **(B)** S-plot generated from OPLS-DA. Triangle represents individual metabolite, where potential biomarkers are highlighted with blue, which is greater than or equal to 0.05 and 0.5 for absolute value of covariance *p* [1] and correlation *p*(corr) [1], respectively. **(C)** The scatter plot of biomarkers in data **(B)**. **(D)** iPath analysis. Blue and yellow lines represent decreased and increased metabolisms, respectively. Out of 49 significant metabolites (*p* < 0.05), 46 were submitted to the online website (https://pathways.embl.de).

The PCA is an unsupervised pattern recognition approach that allows the identification of variables and biomarkers. Thus, these samples were analyzed with orthogonal partial least squares discriminant analysis (OPLS-DA) model. Component t [1] separated the PA-R_SCF_ from PA-S (Figure 4A). Discriminating variables were present in the S-plot, when cutoff values were set as greater than or equal to the 0.05 and 0.5 for the absolute value of covariance *p* [1] and correlation *p* (corr) [1], respectively. A total of 16 biomarkers (adenine, palmitic acid, stearic acid, citric acid, glycine, octadecenoic acid, fructose, GABA, glutamic acid, leucine, succinic acid, valine, ornithine, proline, putrescine, and mannose) were identified (Figure 4B). Abundance of these biomarkers is listed in Figure 4C. Among them, succinic acid and citric acid belong to the P cycle. These results suggest that the reduced P cycle is a characteristic feature in PA-R_SCF_.

The global metabolic fluxes of PA-R_SCF_ were further analyzed with iPath 3.0 that provides a global overview of the metabolomic change. Significantly, the repression of metabolism was dominant (Figure 4D), suggesting a repressed global metabolism in PA-R_SCF_. Logically, the central carbon metabolism should be responsible for this, since it is composed of the flow of carbon from nutrients into biomass and thereby plays a crucial role in metabolic network (Zhang Z. et al., 2020). Indeed, whole reduction was determined in the TCA cycle/the P cycle (Figure 4D). These results indicate the reduced global metabolism in the resistance, where the P cycle plays a role.

### The Central Carbon Metabolisms Are Affected

**FIGURE 5:**
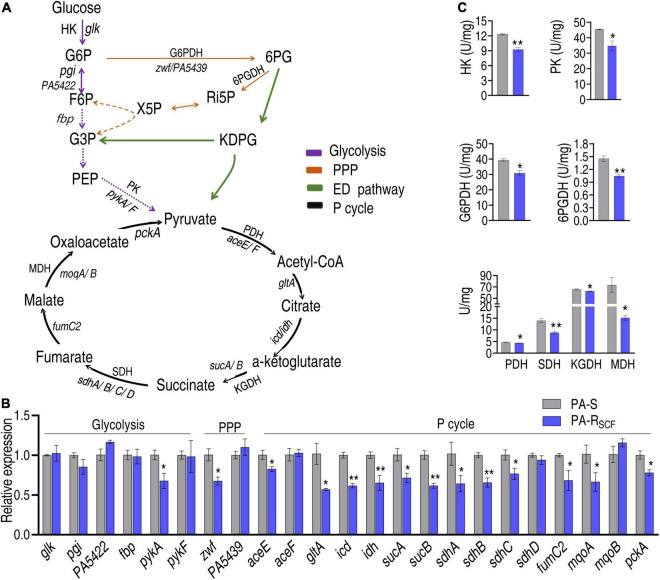
Validation of the depressed central carbon metabolism. **(A)** Map showing that the composition of central carbon metabolism includes glycolysis, PPP, ED pathway, and P cycle. G6P, glucose-6-phosphate; F6P, fructose-6-phosphate; 6PG, 6-phosphogluconate; KDPG, 2-keto-3-deoxy-6-phosphogluconate; Ri5P, ribulose-5-phosphate; X5P, xylulose-5-phosphate; G3P, glyceraldehyde-3-phosphate; PEP, phosphoenolpyruvate; HK, hexokinase; PK, pyruvate kinase; PDH, pyruvate dehydrogenase; KGDH, alpha-ketoglutarate dehydrogenase; SDH, succinate dehydrogenase; MDH, malate dehydrogenase; G6PDH, glucose 6-phsophate dehydrogenase; 6PGDH, 6-phosphogluconate dehydrogenase. Solid lines represent single-step reaction and dashed lines represent multistep reaction. Arrows indicate the direction of the reaction. **(B)** qRT-PCR for expression of key genes in glycolysis, PPP, and P cycle. **(C)** Activity of enzymes in glycolysis, PPP, and P cycle. Results are displayed as mean ± SEM and three biological repeats are performed. Significant differences are identified. *p < 0.05 and **p < 0.01.

To validate the conclusion that the central carbon metabolism is depressed, qRT-PCR and enzyme activity measurement were used to examine the central carbon metabolism. The central carbon metabolism includes glycolysis, pentose phosphate pathway (PPP), and the P cycle (Figure 5A), where ED pathway is indicated since PA is known to mainly use the ED pathway (Kohlstedt and Wittmann, 2019). Out of 6, 2 and 15 genes encoding glycolysis, PPP, and the P cycle, respectively, 1, 1, and 12 were reduced and the other genes were unchanged in PA-R_SCF_ (Figure 5B). Furthermore, activity of HK and PK in glycolysis, of G6PDH and 6PGDH in PPP, and of PDH, KGDH, SDH, and MDH in the P cycle was measured in PA-R_*SCF*_ and PA-S. Lower activity was determined in PA-RSCF than PA-S (Figure 5C). These results validate the downregulated glycolysis, P cycle, and PPP.

### The Central Carbon Metabolism-Related Metabolic Pathways Are Affected

**FIGURE 6:**
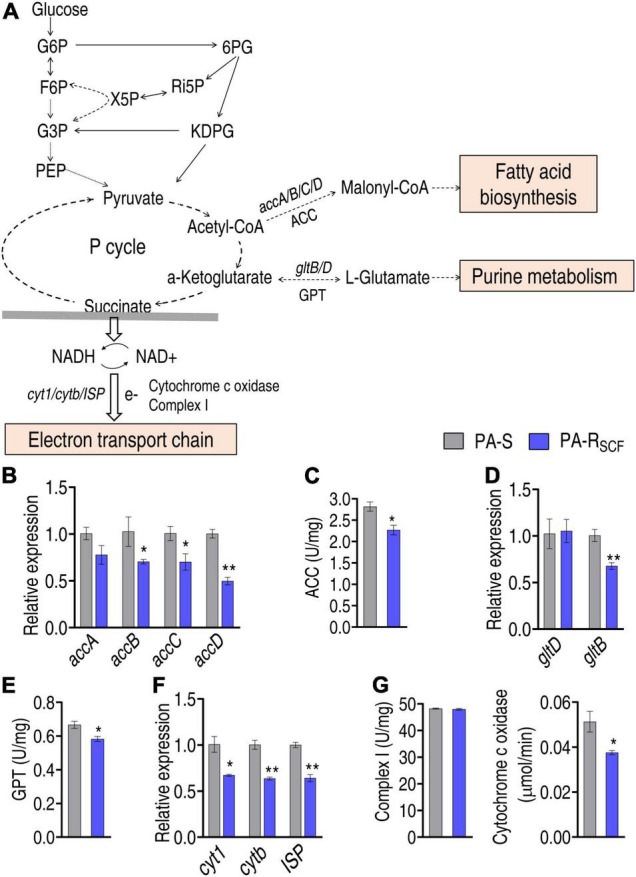
Investigation of the central carbon metabolism-related metabolic pathways. **(A)** Map showing main central carbon metabolism-related metabolic pathways. The abbreviations are referred in Figure 5A legend. Solid lines represent single-step reaction and dashed lines represent multistep reaction. Arrows indicate the direction of the reaction. **(B)** Expression of key genes entering biosynthesis of fatty acids. **(C)** Activity of enzyme entering biosynthesis of fatty acids. ACC, acetyl-CoA carboxylase. **(D)** Expression of key genes entering glutamate metabolism. **(E)** Activity of enzyme entering glutamate metabolism. GPT, glutamic-pyruvic transaminase. **(F)** Expression of key genes entering electron transport chain. **(G)** Activity of enzyme entering electron transport chain. Results are displayed as mean ± SEM and three biological repeats are performed. Significant differences are identified. *p < 0.05 and **p < 0.01.

The central carbon metabolism fluxes to fatty acid synthesis, glutamate metabolism, and electron transport chain, mediated by the P cycle (Figure 6A). Therefore, it is interesting to know how the three metabolic pathways are affected. First, fatty acid synthesis was investigated by quantifying the expression of *accA/B/C/D* that encodes acetyl-CoA carboxylase (ACC) and enzymatic activity of ACC. ACC is a biotin carboxylase that catalyzes the ATP-dependent condensation of acetyl-CoA and carbonate to form malonyl-CoA, the first step for fatty acid synthesis. qRT-PCR showed that the expression of *accB/C/D* was reduced in PA-R_SCF_ compared with that of PA-S (Figure 6B). Consistently, the activity of ACC was lower in PA-R_SCF_ than that of PA-S (Figure 6C). Then, the expression of *gltD* and *gltB*, which encode glutamate synthases, and activity of glutamic-pyruvic transaminase (GPT) were quantified. Lower expression of *gltB* and lower activity of GPT were detected in PA-R_SCF_ than in PA-S (Figures 6D,E). Finally, a similar approach was used to investigate the effect of SCF on expression of *cyt1*, *cytb*, *ISP*, and activity of complex I and cytochrome c oxidase that conveys electron to electron transport chain. The expression of the three genes and the activity of cytochrome c oxidase were reduced in PA-R_SCF_ (Figures 6F,G). These results indicate that SCF resistance downregulates the three main metabolic pathways related to the central carbon metabolism.

### H_2_O_2_ Promotes SCF-Mediated Killing Efficacy

**FIGURE 7:**
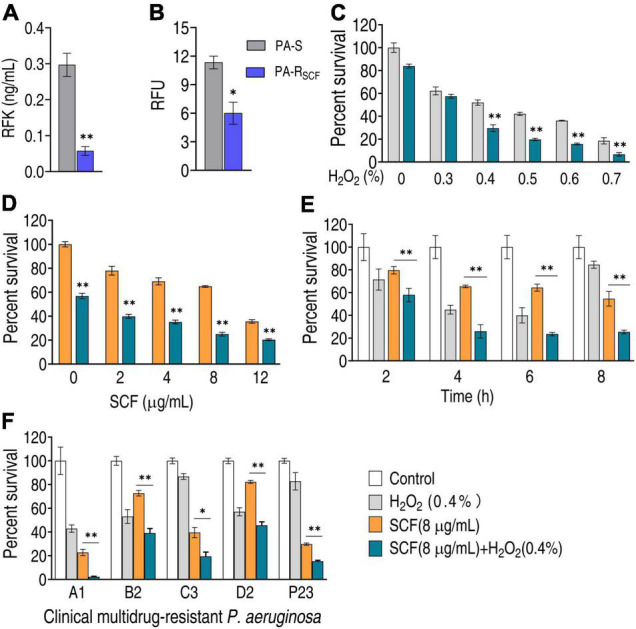
Effect of H_2_O_2_ on bacterial viability in the presence of SCF. **(A)** Activity of riboflavin kinase (RFK) in PA-R_SCF_. **(B)** ROS production in PA-R_SCF_. RFU, relative fluorescence units. **(C)** Percent survival of PA-R_SCF_ in the presence of different concentrations of H_2_O_2_ plus SCF. **(D)** Percent survival of PA-R_SCF_ in the presence of different concentrations of SCF plus H_2_O_2_. **(E)** Percent survival of PA-R_SCF_ in the presence of the indicated time plus SCF and H_2_O_2_. **(F)** Percent survival of clinically multidrug-resistant *P. aeruginosa*. Results are displayed as mean ± SEM and three biological repeats are performed. Significant differences are identified. *p < 0.05 and **p < 0.01.

Besides the three main related metabolic pathways, the central carbon metabolism also fluxes to riboflavin metabolism *via* PPP. RFK plays a crucial role in riboflavin metabolism. The activity of RFK was reduced in PA-R_SCF_ (Figure 7A). Moreover, riboflavin metabolism was selected for further investigation since this metabolic pathway is used for ROS production. Lower ROS was detected in PA-R_SCF_ than in PA-S (Figure 7B). If the reduction contributed to the resistance, bacterial sensitivity to SCF should be increased when ROS is elevated. Percent survival of the pathogen was reduced with increasing H_2_O_2_ concentrations (Figure 7C). Viability was also decreased in different SCF doses (Figure 7D). With the incubation time increasing, the killing was elevated and reached the top at 6 h (Figure 7E). The potentiation was effective to clinically isolate multidrug-resistant *P. aeruginosa* (Figure 7F). These results indicate that SCF resistance is related to the downregulation of riboflavin metabolism in *P. aeruginosa*.

## Discussion

Metabolism plays roles not only in anti-infective immunity against bacterial infection but also in bacterial antibiotic resistance (Peng et al., 2015; Jiang et al., 2019, 2020b; Zhang S. et al., 2020; Yang et al., 2021a,b; Zhao et al., 2021). Therefore, new strategies to combat antibiotic-resistant *P. aeruginosa* infection can be based on metabolism (Dolan, 2020; Kuang et al., 2021b). The present study adopts a metabolomic approach to investigate SCF resistance from a metabolic perspective and identifies the repressed global metabolism as the most characteristic feature of SCF resistance. Therefore, the boosting of the repressed metabolism can be an effective way to control infection caused by SCF-resistant *P. aeruginosa*, which is supported by our very recent report on the NO-potentiated sensitivity to SCF (Kuang et al., 2021b).

The central carbon metabolism is the core component of cell metabolism to convert nutrients into metabolic precursors for biomass and energy to sustain life (Wang et al., 2013; Zhang Z. et al., 2020; Pham et al., 2021). Thus, the present study not only examines the decreased glycolysis, the PPP, and the P cycle of the central carbon metabolism, but also explores the effect on its related metabolic pathways, biosynthesis of fatty acids, glutamate metabolism, and electron transport chain and riboflavin metabolism, where biosynthesis of fatty acids, glutamate metabolism, and electron transport chain are fueled by the P cycle. However, reports have indicated that the P cycle is reduced, but other related metabolic pathways may be enhanced (Liu et al., 2019; Su et al., 2020, 2021). For example, a disrupted pyruvate cycle and decreased energy metabolism but increased fatty acid biosynthesis was identified as a feature in ciprofloxacin-resistant *E. tarda* and ceftazidime-resistant *Vibrio alginolyticus* (Liu et al., 2019; Su et al., 2020). When the increased fatty acid biosynthesis is inhibited, quinolone-mediated killing efficacy to antibiotic-resistant bacteria was elevated in ciprofloxacin-resistant *E. tarda* (Su et al., 2021). Glutamate metabolism and the PPP fuels purine metabolism (Tozzi et al., 2006; Zhao et al., 2021). Metabolic adaptations include increases in acetogenesis, carbon flow through the PPP, wall teichoic acid and peptidoglycan precursor biosynthesis, and purine biosynthesis, but a decrease in TCA cycle activity in vancomycin-intermediate *Staphylococcus aureus* (Gardner et al., 2017). Downregulated central carbon metabolism and nucleotide metabolism were linked to the elevated levels of glycerophosphocholine in carbapenem-resistant *Klebsiella pneumoniae* after exposure to imipenem (Low et al., 2018). Thus, it is interesting to know the effect of the repressed central carbon metabolism on these pathways that are investigated in this study. Decreased gene expression and/or enzyme activity is determined in these metabolic pathways in PA-R_SCF_. Thus, the relationship between the central carbon metabolism and its related metabolic pathways may be different for different bacterial species and antibiotic classes. In the present case, the distribution of the central carbon metabolism to the related metabolic pathways is altered, which contributes to SCF resistance.

To demonstrate that the altered or affected metabolism is related to antibiotic resistance, the present study explores whether the reduced riboflavin metabolism is enhanced to promote SCF-mediated killing efficacy. To demonstrate this, H_2_O_2_ is used. H_2_O_2_ potentiates SCF-mediated killing to both lab-evolved and clinically isolated *P. aeruginosa* strains. Therefore, the repressed central carbon metabolism may support SCF resistance through the altered distribution to the related metabolic pathways such as riboflavin metabolism. The finding that H_2_O_2_ potentiates SCF-mediated killing suggests that SCF is an antibiotic enhanced by ROS.

In addition, recent evidence shows that the P cycle rather than the TCA cycle provides respiratory energy in bacteria (Su et al., 2018). The finding of the P cycle is based on *E. tarda* and *E. coli* with a proof of principle for *Vibrio anguillarum*, *V. alginolyticus*, *V. parahaemolyticus*, *V. vulnificus*, *V. fluvialis*, and *Photobacterium damsel* (Su et al., 2018). Very recently, a GC-MS-based ^13^C metabolic flux analysis is developed to resolve the parallel and cyclic glucose metabolism of *Pseudomonas* PAO1 and *Pseudomonas aeruginosa putida* KT2440 (Berger et al., 2014; Kohlstedt and Wittmann, 2019). The analysis shows that the P cycle occurs in the two strains *via* two ways, oxaloacetate-phosphoenolpyruvate-pyruvate-AcCoA-citrate and malate-pyruvate-AcCoA-citrate, to connect the TCA to the P cycle. Interestingly, the major flux is from phosphoenolpyruvate to pyruvate to AcCoA, with appreciable amount of carbon going to oxalacetate from pyruvate (Kohlstedt and Wittmann, 2019), which is consistent with oxaloacetate instead of AcCoA as a fuel for the P cycle (Su et al., 2018). The repressed P cycle (including the TCA cycle) is related to antibiotic resistance and has been reported in some bacteria including *Vibrio alginolyticus*, *Edwardsiella tarda*, and *Stenotrophomonas maltophilia* (Cheng et al., 2018; Gil-Gil et al., 2020; Li et al., 2020; Su et al., 2020; Ye et al., 2021). Our recent report shows that the P cycle is downregulated (Kuang et al., 2021b). However, the repressed central carbon metabolism as a characteristic feature is not examined in these bacteria. The present study demonstrates that the central carbon metabolism is downregulated, which exerts effect on the resistance *via* influencing downstream metabolic pathways.

## Conclusion

The present study reveals that the global metabolism is repressed in SCF-resistant *P. aeruginosa*. Among the altered metabolic pathways, the repressed central carbon metabolism is a characteristic feature in SCF resistance. The repressed central carbon metabolism affects the related metabolic pathways, where the reduced riboflavin metabolism contributes to SCF resistance. These results highlight the way to understand SCF-induced resistance mechanism from a metabolic perspective.

## Data Availability Statement

The original contributions presented in the study are included in the article/Supplementary Material, further inquiries can be directed to the corresponding author/s.

## Author Contributions

Z-GC and HL conceptualized, designed the project, and wrote the manuscript. Z-GC, HL, and X-XP interpreted the data. Y-TC, K-XY, Z-YD, and HY performed the experiments. Y-TC performed the data analysis. All authors contributed to the article and approved the submitted version.

## Conflict of Interest

The authors declare that the research was conducted in the absence of any commercial or financial relationships that could be construed as a potential conflict of interest.

## Publisher’s Note

All claims expressed in this article are solely those of the authors and do not necessarily represent those of their affiliated organizations, or those of the publisher, the editors and the reviewers. Any product that may be evaluated in this article, or claim that may be made by its manufacturer, is not guaranteed or endorsed by the publisher.

## Footnotes

1
https://www.metaboanalyst.ca/

2
https://pathways.embl.de/
