# Supplementary material for: Repressed Central Carbon Metabolism and Its Effect on Related Metabolic Pathways in Cefoperazone/Sulbactam-Resistant Pseudomonas aeruginosa
Source: Front Microbiol. 2022 Mar 3;13:847634. doi: 10.3389/fmicb.2022.847634 (PMC8927769; doi:10.3389/fmicb.2022.847634)
Supplement: Supplementary file 1 [file Table_1.DOCX]

| **Supplementary Table S1** Primers for qRT-PCR | | | |
| --- | --- | --- | --- |
| Gene | Primer (5'-3') | Gene | Primer (5'-3') |
| 16SrRNA-F | CAAAACTACTGAGCTAGAGTACG | sdhB-F | CAAGGAACAGGACGAGGGC |
| 16SrRNA-R | TAAGATCTCAAGGATCCCAACGGCT | sdhB-R | AGAAGGACGGGCAGGAGGT |
| glk-F | TAATGTAGACGCCACCGAGC | sdhC-F | CCGTGAATAGCAAACGACCTG |
| glk-R | CCTGCTCGCCCTCTATGAAA | sdhC-R | GGCAATACCGAGGAACAGGA |
| pgi-F | CCGCAACTGGTATCCGAAGC | sdhD-F | TTCCTGCTGGGCTACCTCATT |
| pgi-R | GGAGGAAACGATGAACAGGGTG | sdhD-R | CAGGGTCAACAGGCTGAAGAT |
| PA5422-F | AGCGCAACCCGCAGGAAG | fumC2-F | GGACCGAAACCGATAGCC |
| PA5422-R | GCGGAAGCTGACGCAGAGGT | fumC2-R | GCGGGAAGTGTTCGTCGTG |
| fbp-F | TCCTGCTGGAAGCCAACGA | mqoA-F | CCAATGGCGACAAGGAAAC |
| fbp-R | ATGGTGCCGACCGAGACG | mqoA-R | CGGACCGAACAGCAGAACC |
| pykA-F | AAGTTCCGCTTCTCCACCAG | mqoB-F | CCCCTATGCCGGTTTCTCC |
| pykA-R | CTTGATGTCGGCCTTGTCCT | mqoB-R | CGGGTCAGGTCCATGTTGT |
| pykF-F | TTCGCCATCATCTCTACCGC | pckA-F | GCCCAGGACCATTTCGTTT |
| pykF-R | GCATCCAGAAACGCATCGTC | pckA-R | GCCGAAGAACAGGGTCACAT |
| zwf-F | GGACCTTGACCTTCTCGTCG | accA-F | CCTGCAAGCCAAGATCGAAGAG |
| zwf-R | CGGATCGACCATTACCTGGG | accA-R | CCGATGTAGTCGAGGGTATAGGGA |
| PA5439-F | CGACAACGACAGCGATACCG | accB-F | GAAGTCGGCCAGAGCGTGA |
| PA5439-R | GGATGACGATTTCCGAGCACTT | accB-R | AGCGGCTGGTCGAACTCAA |
| aceE-F | CAGTTCCCCACCGTATCCA | accC-F | CTCGCGTGCAGGTGGAGC |
| aceE-R | CGAAGATCAGGTTGTCGAGTTT | accC-R | TCGGGTCTTCGGCGTTGA |
| aceF-F | GGCCCGCAACAAGAAGCT | accD-F | CCTGGATGTCTGCCCGAAGT |
| aceF-R | CTTGCCGTCCCATACCG | accD-R | GGCGAGGCGATCCTTGTACT |
| gltA-F | CGTGATGTGCGGCGTGA | gltB-F | CGGAAACCCACCAGACCCT |
| gltA-R | GGTCGGCGTGGAGAATGA | gltB-R | CGCAGTTGTTCAGATGGCAGAT |
| icd-F | CCGAGGAAATGGGCGTCA | gltD-F | GCATGGGCATCGAGTTTCG |
| icd-R | CGACTTCGTAGCCCCAATC | gltD-R | GAAGTCCAGGGCGTCGTGTA |
| idh-F | GAACCCGAACAACGGTATCTC | cyt1-F | GCTGCAAGCAGGTACAGGTGG |
| idh-R | CCGCCTTGGTGTCTTTCTGCT | cyt1-R | GCGAATACGAAGAAGAAGGCC |
| sucA-F | CGCCAAGCAGCGTACTACCC | cytb-F | AACCAGTTCAAGACCCCCG |
| sucA-R | GGCGAAGCCCCAGTTGA | cytb-R | AGCCAGCCCTTGTAGCGGA |
| sucB-F | GTGGCGTGTTCGGTTCCCT | ISP-F | GCGGCAAGCCTGTATTCATC |
| sucB-R | ATCAGACGGTGGTCGTAGGA | ISP-R | CGTGGCAAGGGCAGAAGT |
| sdhA-F | GAAGAAGTCGCTCCGCTCAA |  |  |
| sdhA-R | GAATGCCTGGCTCTTGTCG |  |  |
